# Magnetic resonance imaging of fibroadenoma-like lesions and correlation with Breast Imaging-Reporting and Data System and Kaiser scoring system

**DOI:** 10.4102/sajr.v22i2.1532

**Published:** 2018-11-07

**Authors:** Daniel J. Cloete, Cornelia Minne, Peter K. Schoub, Jan H.R Becker

**Affiliations:** 1 Dr George Mukhari Academic Hospital, Ga-Rankuwa, South Africa; 2 Department of Diagnostic Radiology, Sefako Makgatho Health Sciences University, South Africa; 3 Department of Radiology, Parklane Radiology, Johannesburg, South Africa; 4 Department of General Surgery, Sefako Makgatho Health Sciences University, South Africa

## Abstract

**Background:**

Multiple breast lesions resembling fibroadenomas are a common imaging finding in patients presenting to the mammography unit at Dr George Mukhari Academic Hospital in the North-West district of Tshwane, South Africa. Patients often present with multiple lesions, up to 20 lesions per breast. These lesions often have atypical features on ultrasound and/or a clinical history of growth is commonly given. Phyllodes tumours may be indistinguishable from fibroadenomas and breast cancers may on occasion present with benign features, which can lead to misdiagnosis. Breast magnetic resonance imaging (bMRI) evaluation of lesions resembling fibroadenomas may improve accurate assessment and identification of lesions requiring biopsy.

**Objectives:**

To assess the reliability of bMRI to characterise lesions resembling fibroadenomas on ultrasound, using the Breast Imaging-Reporting and Data System (BI-RADS) and Kaiser scoring systems with histopathological correlation.

**Method:**

A quantitative, prospective, investigative study was performed with a sample size of 100 breast lesions among a total of 35 patients at Dr George Mukhari Academic Hospital. Patients were recruited after a breast ultrasound investigation revealed lesions resembling fibroadenomas, but with an indication for ultrasound-guided biopsy, for example, very large size, atypical features on ultrasound or a history of recent growth. The bMRI was performed prior to the ultrasound-guided breast biopsies. Three investigators independently evaluated the bMRI and applied BI-RADS descriptors to each lesion. The Kaiser score was then calculated for each lesion. Statistics were calculated using Pearson’s and Spearman’s coefficients for inter-reader variability, kappa scores for BI-RADS and Kaiser score correlation with histology.

**Results:**

Evaluation with bMRI, BI-RADS and the Kaiser scoring system showed statistically significant correlation with each other and with histopathology results for each lesion. There was statistically significant agreement among the investigators regarding the interpretation of the lesions and allocation of appropriate BI-RADS scores.

**Conclusion:**

Multiple lesions resembling fibroadenomas can be evaluated with bMRI when multiple breast biopsies would not be feasible. With a good imaging protocol and technique, adequate interpretation skills by the radiologist and the use of the Kaiser scoring system, an accurate diagnosis can be achieved.

## Introduction

Fibroadenomas of the breast are one of the most common benign masses female patients present with to their health care practitioner. It affects women mostly of childbearing age and is a cause of concern for these patients, especially in the era of breast cancer awareness.^1^ Some patients present with multiple and/or rapidly growing lesions.^1^ These findings warrant further investigation, resulting in referral to the radiologist. Recent research is pointing towards safer imaging methods with reliable and reproducible results aiming to be more acceptable to patients.

Fibroadenomas comprise epithelial and stromal components. Their growth patterns are described as intra-canalicular or peri-canalicular.^2^ Fibroadenomas are abnormalities of normal development and involution of breast tissue and form part of the broader group of fibroepithelial lesions. Different varieties of fibroadenomas exist, namely, giant, multiple successive, juvenile fibroadenoma and fibroadenoma in pregnancy and lactation. The malignant, recurring counterpart of the fibroadenoma, is the phyllodes tumour.^3^ Although a small increase in the size of a fibroadenoma may be observed towards the end of each menstrual cycle because of hormonal responses and the presence of oestrogen and progesterone receptors, rapid growth of a lesion may be indicative of a breast cancer or phyllodes tumour.^2^ Phyllodes tumours are considered rare; however, a higher incidence is observed among Latin Americans and Asians.^4^ Tan et al. reported seven phyllodes tumours for every 100 breast cancers diagnosed.^3^ Another cohort from Turkey investigated 72 phyllodes tumours that were diagnosed and treated over a 3-year time period and compared to fibroadenomas. They found significant atypical features on ultrasound and breast magnetic resonance imaging (bMRI) in phyllodes tumours, as compared to fibroadenomas.^5^

Patients with a family history of breast cancer, previous or current personal history of breast cancer, complex fibroadenomas or the presence of histological atypia have a higher risk for breast cancer. Although there is still controversy in the literature whether fibroadenomas can undergo malignant transformation, some researchers opine that it is possible. The epithelial component of a fibroadenoma may develop a carcinoma *in situ* (ductal carcinoma *in situ* [DCIS]) or an invasive carcinoma.^6^ It is also known that fibroadenomas can co-exist with carcinomas. Wu et al. reported lesions with a benign appearance on imaging that proved to be invasive ductal carcinoma with ipsilateral nodal metastases.^7^

**FIGURE 1:**
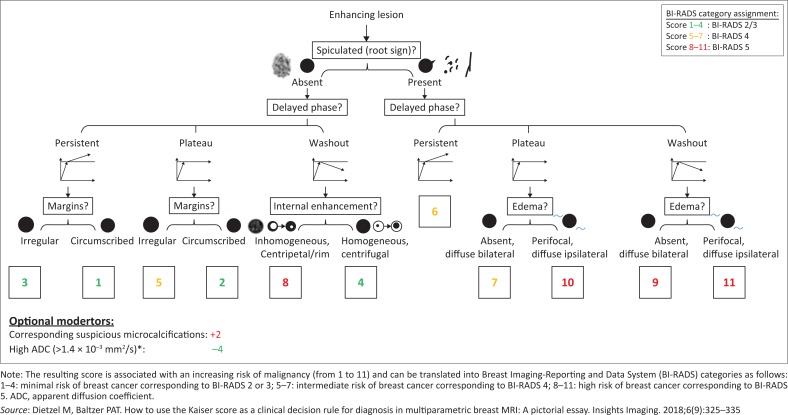
Kaiser score flowchart*: The Kaiser score is assigned by following a simple flowchart from the top to the bottom, which lets the reader assign the presence or absence of four diagnostic criteria.

The Breast Imaging-Reporting and Data System (BI-RADS) was developed to standardise nomenclature and recommendations used by radiologists. In addition, the recently developed Kaiser score was put to the test in this research project to evaluate how it compares in accuracy with the BI-RADS allocations for MRI. The Kaiser scoring system was developed to assist radiologists to accurately and consistently determine the BI-RADS score of each lesion, using bMRI characteristics, with respect to the morphology, dynamic contrast enhancement and diffusion-weighted imaging (DWI) features of the lesion.^8^ It also includes mammographic findings (if available), specifically the presence of micro-calcifications. A BI-RADS score is then allocated according to the number of Kaiser points allocated (Figure 1 and Table 1).^8^

**TABLE 1:** Kaiser score categories with typical benign and malignant histopathological correlates.

Kaiser score	Benign	Malignant
1	Fibroadenoma	n/a
2	Fibroadenoma, adenosis, papilloma	IDC
3	Benign epithelial proliferations, inflammatory changes	DCIS
4	Adenosis	IDC
5	Benign and atypical epithelial proliferations, fibroadenoma, fibroadenomatoid hyperplasia, papillomatosis	DCIS, IDC
6	Scar tissue and inflammation	IDC, ILC
7	Scar tissue and inflammation	IDC, ILC
8	Atypical fibroadenoma, adenosis proliferative	High IDC, metastasis, lymphoma
9	n/a	IDC, ILC
10	n/a	IDC, ILC
11	n/a	IDC, ILC

*Source*: Used with permission from Dietzel M, Baltzer PAT. How to use the Kaiser score as a clinical decision rule for diagnosis in multiparametric breast MRI: A pictorial essay. Insights Imaging. 2018;6(9):325–335

DCIS, ductal carcinoma *in situ*; IDC, invasive ductal carcinoma; ILC, invasive lobular carcinoma; n/a, not applicable.

## Methods

A quantitative, prospective, investigative study was performed with a sample size of 100 breast lesions among a total of 35 patients at Dr George Mukhari Academic Hospital. Patients with breast masses who were referred to the mammography unit of Dr George Mukhari Academic Hospital in the North-West district of Tshwane, South Africa, between 01 July 2015 and 28 February 2017, were considered for the study.

Patients were recruited after a breast ultrasound investigation revealed lesions resembling fibroadenomas but with an indication for ultrasound-guided biopsy, for example, very large size, atypical features on ultrasound, a history of recent growth, age of first presentation above 35 or patient anxiety. Patients were enrolled on a voluntary basis and consent was signed prior to workup. Study participants were informed that they could withdraw from the research project at any time, without negatively impacting their further workup and management.

Breast ultrasound was performed on all patients with a Philips Epiq 5 ultrasound machine. Breast Imaging-Reporting and Data System scores were allocated to each lesion according to the ultrasound appearance. The ultrasound examinations were performed by the radiology residents and/or general radiologist on rotation in mammography at the time of the patient’s initial presentation. Patients aged 40 and above also had a mammogram included in their workup.

Breast magnetic resonance imaging was acquired on a 1.5 T machine (Toshiba Vantage or Philips Multiva) using a dedicated double breast coil. All patients were examined in the prone position using an identical protocol. The bMRI protocol included T2-weighted (T2W) and T2-weighted fat saturation (T2W FS) sequences in the axial orientation. Diffusion-weighted imaging and apparent diffusion coefficient (ADC) values were calculated using *b*-values of 0 and 800. Diffusion-weighted imaging sequences were performed before the dynamic contrast-enhanced (DCE) imaging sequences (see Table 2). The dynamic study was obtained using a T1-weighted fat-saturated (T1W FS) sequence in the axial plane. After acquisition of the native T1W FS scan, gadopentetate dimeglumine (Magnevist, Schering, 0.1 mmol/kg of body weight) was administered intravenously as a rapid bolus (4 mL/s), followed by a 20 mL saline flush at a rate of 4 mL/s (see Table 1 for the dynamic scan parameters). Following the contrast bolus and saline administration, DCE commenced after a 75 s delay and continued with the same sequence parameters and under identical tuning conditions at 1-min intervals for 6 min. Post-processing of the dynamic study included the calculation of time–signal intensity curves and subtraction of unenhanced images from the contrast-enhanced dynamic images.

**TABLE 2:** Dynamic T1-weighted fat saturation scan parameters.

Parameters	Variable
TR/TE	7/4.0
Flip angle	90°
Slice thickness	1.5 mm
Gap	0.6 mm
Field of view	340 mm × 340 mm^2^
Resolution	1.37 mm × 1.37 mm
Orientation	axial
Slices	24

TR, repetition time in milliseconds (ms); TE, echo time in milliseconds (ms).

Ultrasound-guided core needle biopsies were performed after the bMRI. Histology specimens were examined and reported by qualified National Health Laboratory Services (NHLS) pathologists based at Sefako Makgatho Health Sciences University. The histopathology results were captured on a datasheet and later compared with the allocated BI-RADS scores for each lesion.

The MRI studies were interpreted by three independent readers, who were blinded to the histology results and clinical data of each patient. Two of the readers have 10 years of experience each in general radiology, with a special interest in breast imaging, and one reader was a radiology resident with 10 months mammography experience. The data were interpreted on a Hologic Multiview workstation with Multiview MM version 4.1 software. This programme uses the MRI BI-RADS format for reporting. Breast Imaging-Reporting and Data System scores were allocated to each lesion as interpreted by each reader.

Lesion size (two dimensions on axial image and one dimension on coronal image), location (clock position, quadrant of breast, left or right breast and whether axilla area or central breast area or retro areola area), morphology (circumscribed or non-circumscribed margins), shape (round or oval), enhancement pattern (persistent, plateau or washout) and diffusivity were recorded.

**FIGURE 2:**
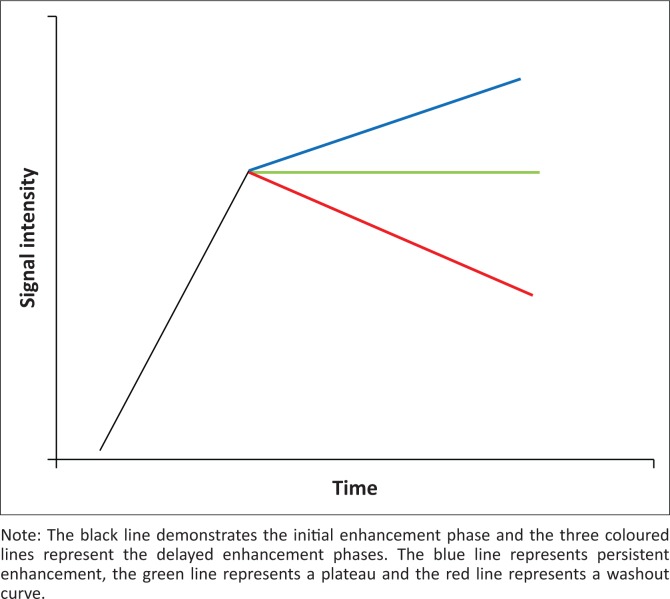
A diagram demonstrating the signal intensity–time curves.

The degree of enhancement determined at three (or more) time points during the dynamic bMRI was used to draw a kinetic curve that reflected the vascularity of the lesion. Three types of kinetic curves are in use (Figure 2):

- Type 1 (persistent) curves: generally, 95% of lesions with this type curve are benign.
- Type 2 (plateau) curves are often equivocal; 50% of lesions with this curve will be malignant.
- Type 3 (washout) curves are predictors of malignancy, with a malignant rate of 90%.^8,9^

The Kaiser clinical scoring system was used to allocate points that were translated to a BI-RADS score for each lesion. The BI-RADS scores according to the Kaiser scoring system were captured on a datasheet and compared to the reader’s BI-RADS allocations and histopathology results for each lesion.^8^ All data were transferred to a spreadsheet and interpreted by a statistician on the SAS programme version 9.3 running under Microsoft Windows.

## Ethical consideration

Approval was received from the institutional ethics review board and permission to conduct the study was obtained from the CEO and hospital management (Ethics number SMUREC/M/15/2015: PG). Participants were informed of the purpose of the study and examinations that would be performed. Participation was voluntary, but without negatively impacting their management.

## Results

The age of the 35 patients ranged from 16 to 57 years, with a mean age of 28.5 (±11, 51 standard deviation [SD]) and a median age of 24 years (20–39 interquartile ratio [IQR]).

### Lesion characteristics

#### Margin

**FIGURE 3:**
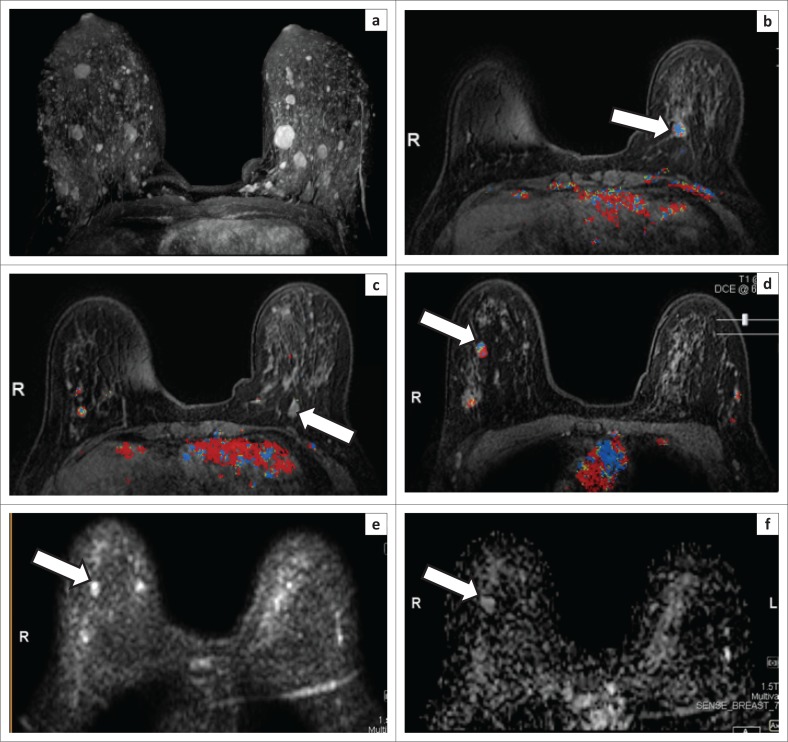
(a) A maximum intensity projection image of the T1-weighted fat saturation post-contrast sequence of a patient with a proven intermediate phyllodes tumour in the right breast demonstrating multiple bilateral breast lesions. (b–d) The dynamic contrast-enhanced images of the same patient demonstrating multiple lesions with varying enhancement patterns. The superimposed colour map on each image reflects the enhancement curves of the pixels. Pixels in red indicate areas with a washout curve, green a plateau curve and blue a persistent enhancement curve. The lesion demonstrated with an arrow in (b) shows a typically persistent enhancement curve. The lesion indicated with an arrow in (c) did not demonstrate any enhancement. The lesion shown with an arrow in (d) had a predominant washout curve and was proven to be a phyllodes tumour. (e) Diffusion-weighted imaging demonstrating restricted diffusion in the phyllodes tumour (arrow). (f) The apparent diffusion coefficient (ADC) map for the phyllodes tumour (arrow) demonstrated a low ADC value of 1.33 mm^2^/s.

One fibroadenoma and one papilloma had a partially circumscribed margin. All the other lesions had circumscribed margins, including the two phyllodes tumours (Figure 3).

#### Shape

As far as shape was concerned, three lesions had irregular shapes, 12 lesions were lobular, 72 lesions were oval and 13 lesions were round.

#### Mass enhancement patterns

Heterogeneous enhancement was observed in 16 lesions, homogeneous enhancement in 77 lesions, rim enhancement in three lesions and dark, non-enhancing internal septations in four lesions.

#### Enhancement curves

In this study, 60% of lesions had type 1 curves (persistent enhancement), 23% of lesions demonstrated type 2 curves (plateau enhancement) and 17% of lesions had type 3 curves (washout).

#### Apparent diffusion coefficient

A total of 19 lesions showed no restriction, 18 lesions demonstrated partial restriction and 63 lesions showed restricted diffusion. The ADC of the intermediate phyllodes was 0.51 mm^2^/s and the benign phyllodes was 1.27 mm^2^/s. The ADC values of 35 fibroadenomas fell within the range 1.41 × 10^−3^ mm^2^/s to 2.01 × 10^−3^ mm^2^/s (see Table 3 for a summary of ADC values for all lesion groups).

**TABLE 3:** Summary of apparent diffusion coefficient values for all the different lesion groups.

Histology	*N*	Mean ADC	Highest	Lowest
Adenoma	3	1.63	2.04	1.04
Proliferative fibrocystic changes	1	1.52	-	-
Benign proliferative changes	2	1.32	1.51	1.14
Fibrous mastopathy	1	1.33	-	-
Fibroadenoma	87	1.33	2.18	0.81
Papilloma	3	1.14	1.44	0.99
Ductal hyperplasia	1	0.98	-	-
Benign phyllodes	1	1.27	-	-
Intermediate phyllodes	1	0.51	-	-

ADC, apparent diffusion coefficient.

### Breast Imaging-Reporting and Data System scores

The bMRI reader BI-RADS assessment of the 100 lesions investigated revealed 75 BI-RADS 2, 21 BI-RADS 3 and four BI-RADS 4 lesions. Histologically, 87 of the lesions were fibroadenomas. A total of 73/87 (83.9%) histologically proven fibroadenomas were scored BI-RADS 2 and 12/87 (13.8%) BI-RADS 3 and 2/87 (2.3%) BI-RADS 5. Two phyllodes tumours received BI-RADS scores of 3 and 4.

Using the Kaiser scoring system changed the BI-RADS allocation of 21 lesions. The Kaiser scoring system upgraded one lesion from BI-RADS 3 to BI-RADS 5 (final histology was fibroadenoma); two BI-RADS 4 lesions were upgraded to BI-RADS 5 (final histology reports were intermediate phyllodes and fibroadenoma) and four BI-RADS 2 lesions were upgraded to BI-RADS 3 (all proven fibroadenomas). Eleven lesions were downgraded from BI-RADS 3 to BI-RADS 2 (all proven fibroadenomas) and three BI-RADS 4 lesions were downgraded to BI-RADS 3 (two fibroadenomas and one lactating adenoma) (see Table 4 for a summary of MRI reader-allocated BI-RADS compared to MRI Kaiser BI-RADS). There was statistically significant correlation between the Kaiser BI-RADS scores and histopathology (*p* < 0.0001), with a confidence interval of 74% – 89%.

**TABLE 4:** Summary of magnetic resonance imaging reader Breast Imaging-Reporting and Data System and magnetic resonance imaging Kaiser BI-RADS.

BI-RADS Allocations	MRI reader BI-RADS			MRI Kaiser BI-RADS			
	BI-RADS 2	BI-RADS 3	BI-RADS 4	BI-RADS 2	BI-RADS 3	BI-RADS 4	BI-RADS 5
Total lesions	75	21	4	83	14	0	3

BI-RADS, Breast Imaging-Reporting and Data System, MRI, magnetic resonance imaging.

### Inter-reader variability

Inter-reader agreement among the three readers was determined to be statistically significant using the McNemar test (*p* = 0.317). Kappa statistics were used to determine agreement between the readers and the Kaiser scoring system. There was good (weighted kappa of 0.73) to very good (weighted kappa of 1.00) strength of agreement demonstrated. There was statistically significant correlation between the MRI reader-allocated BI-RADS and BI-RADS score according to the Kaiser score (*p* < 0.001).

### Sensitivity, specificity, positive predictive values and negative predictive values

The reader-allocated MRI BI-RADS had a sensitivity of 50%, specificity of 76.5%, positive predictive values (PPVs) of 4.17%, negative predictive values (NPVs) of 98.6% and accuracy of 76%. In comparison, the Kaiser-allocated BI-RADS had a sensitivity of 50%, specificity of 84.6%, PPV of 6.25%, NPV of 98.8% and an accuracy of 84%. The differences between the reader and Kaiser-allocated BI-RADS were not statistically significant (*p* = 0.55).

A possible explanation for the low PPV was the absence of malignant lesions and the low number of high-risk lesions like phyllodes tumours. The value of bMRI in detecting breast cancers has been proven in the literature.

## Discussion

Patients with multiple breast lesions resembling fibroadenomas but with atypical ultrasound findings or other indications for biopsy are a common occurrence in daily practice in the South African public health sector. Magnetic resonance imaging could be a valuable tool in identifying lesions that should be biopsied. To appreciate what MRI could contribute to the assessment of the lesions in these patients, it is essential to understand the spectrum of MRI findings of the lesions. Magnetic resonance imaging is an expensive modality and utilisation must be carefully weighed against the risk of missing a breast cancer, phyllodes tumour or other high-risk lesions in a patient with numerous lesions.

Fibroadenomas are classically described as masses that occur in young patients in their reproductive years. This study has confirmed that most patients were in the under 30 age group but patients who were of a more advanced age were also imaged. One of the youngest patients (17 years) in the cohort had a rapidly growing lesion (3.4 cm × 2.6 cm) and had stopped breast feeding 5 months before she presented. Ultrasound demonstrated a lesion with atypical features and a reactive axillary lymph node with borderline abnormal cortical thickness. The lesion biopsied proved to be a lactating adenoma. Breast biopsies are sometimes performed in the institution on anxious patients who request biopsies. This deviation from the BI-RADS guidelines is because the unit often experiences poor patient compliance with follow-up visits because of socio-economic reasons.

**FIGURE 4:**
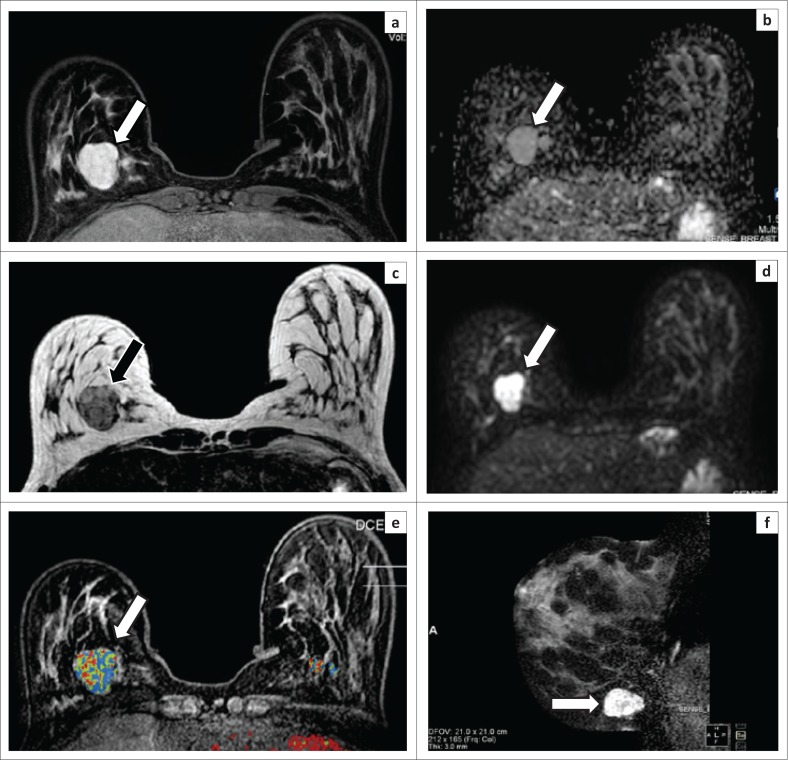
(a–f) demonstrate the appearance of a histologically proven fibroadenoma in the right breast (white and black arrows) on magnetic resonance imaging. (a) A post-contrast T1-weighted fat saturation axial image shows a circumscribed enhancing mass with thin non-enhancing septa. (b) On the apparent diffusion coefficient map, the lesion has an intermediate signal intensity with a value of 1.754 mm^2^/s. (c) A T2-weighted sequence demonstrates a heterogeneous mass that is hyperintense to glandular tissue with hypointense internal septation. (d) The diffusion-weighted imaging image reveals restricted diffusion. (e) The dynamic contrast-enhanced image with a superimposed colour map demonstrates areas of variable enhancement, and the majority of the lesion has a persistent enhancement curve. (f) A sagittal T2-weighted fat saturation image demonstrates a heterogeneously hyperintense lesion.

During bMRI assessment, morphology, margins, enhancement patterns and the kinetic curve are routinely used to allocate a BI-RADS score to each lesion. Most of the lesions in this study were circumscribed and oval, lobular or round, features consistent with those found in the literature for fibroadenomas (see Figure 4 for the typical appearance of benign fibroadenoma on bMRI). Wurdinger et al. described 90% of fibroadenomas in their study having round or lobular shapes.^10^ They also found a smaller frequency of lesions with internal septations. Evaluation of shape and morphology alone was not enough to ascertain benignity of lesions in previous studies.^11^ The same experience was encountered with the study reported here.

Diffusion-weighted imaging is gaining interest in breast imaging. Although restricted diffusion may be a sign of malignancy, the ADC value aids further in differentiating between benign and malignant lesions. The literature reports ADC values of malignant breast tumours ranging from 0.9 × 10^−3^ mm^2^/s to 1.61 × 10^−3^ mm^2^/s.^12^ In this study, the phyllodes tumours had low ADC values. The ADC of the intermediate phyllodes was 0.51 mm^2^/s and that of the benign phyllodes was 1.27 mm^2^/s.

The mean ADC values of normal breast tissue acquired with *b*-values ranging between 0 and 1074 vary from 1.51 × 10^−3^ mm^2^/s to 2.37 × 10^−3^ mm^2^/s. An artificial reduction of ADC values in woman with less dense breasts may occur because of partial voluming with fat tissue. Fibroadenomas would be expected to have high rates of diffusion and ADC values because of stromal myxoid changes and consequently increased mobility of water. However, fibroadenomas with a predominant fibrous component will have lower ADC values.^13^ Papillomas can also have low ADC values because of high cellularity. As in current literature reports, the ADC values of 35 histologically proven fibroadenomas fell within the reported benign range from 1.41 × 10^−3^ mm^2^/s to 2.01 × 10^−3^ mm^2^/s.^12^ These ADC values can be explained by several factors: it may be because of the technique used, density of the patient’s breasts in the study population and the amount of fibrous tissue within the fibroadenomas. This study demonstrated that using ADC in conjunction with other parameters in the Kaiser flowchart increased the accuracy of the assessment.

Another significant observation was that the one patient who had the intermediate phyllodes, had seven benign appearing breast masses (Figure 3). This confirms certain reports in the literature that multifocality does not always equal benignity, and lesions must always be assessed individually to identify potential breast cancer and/or high-risk lesions.^10^

**FIGURE 5:**
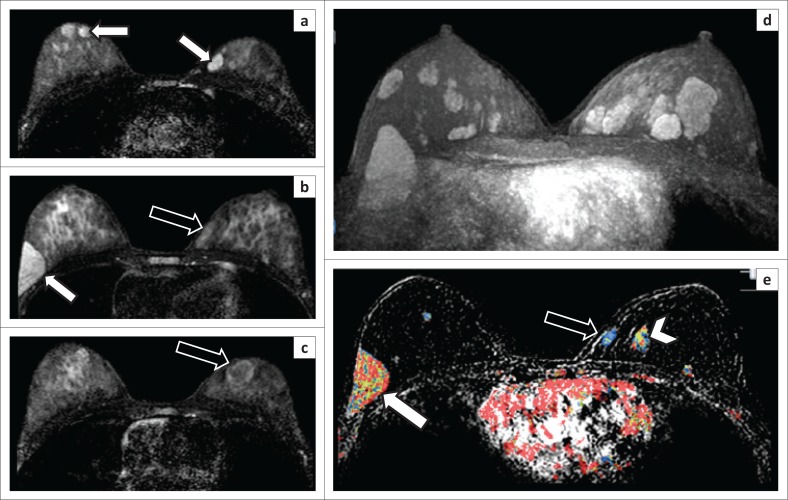
A breast magnetic resonance imaging of a patient with a total of 15 fibroadenomas demonstrating different magnetic resonance imaging appearances: (a–c) The T2-weighted fat saturation images demonstrate multiple homogeneously hyperintense circumscribed oval lesions (white arrows). Two circumscribed hypointense lesions (black arrows) can be seen in (b) and (c). In (d), a maximum intensity projection T1-weighted fat saturation post-contrast image, numerous breast masses are visualised. (e) The subtracted post-contrast T1-weighted fat saturation image with superimposed colour map demonstrates three lesions with different enhancement curves. One lesion with a predominant washout curve (white arrow), a lesion with a persistent enhancement curve (black arrow) and a lesion with mixed enhancement curve (arrowhead).

There was good correlation between the reader MRI BI-RADS and Kaiser scores. Although the accuracy improved slightly when the Kaiser scoring system was employed to allocate BI-RADS scores to lesions. This underlines the value of using structured reporting and the Kaiser scoring system. Most lesions imaged were suspected benign lesions but with atypical ultrasound findings or another indication for biopsy, for example, recent growth, large size or advanced age at presentation. The MRI evaluation confirmed benignity with a high NPV. The indication for bMRI should be based on clinical grounds and multifocality (Figure 5).

## Advantages and limitations

Patients who present with multiple breast lesions that resemble fibroadenomas but with atypical features or other indications for a biopsy, create a dilemma in the workup. There is a paucity of studies addressing this dilemma. This research project specifically targeted this subset of patients. Limitations of the study were the sample size of 100 lesions. A selection bias was created by selecting patients after an ultrasound investigation had revealed lesions resembling fibroadenomas. The study population was selected, however, to specifically study this group of patients. The spectrum of breast disease was limited with no malignant lesions, only two phyllodes tumours and few patients with papilloma.

## Implications and recommendations

Triparametric bMRI should not replace ultrasound and invasive evaluation methods of benign appearing breast lesions in patients with only a few breast lesions, but it is certainly beneficial in patients with multiple lesions (Figure 5).

Triparametric bMRI is recommended for patients with multiple breast lesions, with one or more atypical ultrasound features and age above 35, in whom multiple breast biopsies are not feasible. It will assist in determining whether a biopsy is indicated and to identify which lesions to biopsy. A structured reporting template and the use of the Kaiser scoring system are recommended to improve the accuracy, confidence and reproducibility of the report.

A tailored, well-structured bMRI protocol in the hands of a trained technician can avoid time-consuming and unnecessary long scan times.^9^

## Conclusion

Although well-circumscribed, benign-appearing breast lesions are mostly benign, they cannot always be dismissed as such. Selection of masses for biopsy based on patient history, clinical examination and imaging findings (ultrasound, mammogram and bMRI when indicated) may diminish unnecessary biopsies in patients with multifocal breast lesions. This may be beneficial to reduce patient anxiety and improve compliance.
